# Endobronchial Pulmonary Actinomycosis With Concomitant Tuberculosis Resembling Lung Malignancy on Presentation: A Case Report

**DOI:** 10.1002/rcr2.70813

**Published:** 2026-11-09

**Authors:** Safal Bogati, Bishal Pradhan, Suraksha Thapa, Milesh Jung Sijapati, Prabhas Joshi, Richa Ghimire, Samikchhya Regmi

**Affiliations:** ^1^ Department of Internal Medicine KIST Medical College and Teaching Hospital Lalitpur Nepal; ^2^ Shree Birendra Hospital Kathmandu Nepal; ^3^ Department of Radiology KIST Medical College and Teaching Hospital Lalitpur Nepal; ^4^ Department of Pathology KIST Medical College and Teaching Hospital Lalitpur Nepal

**Keywords:** actinomycosis, case reports, lung cancer, tuberculosis

## Abstract

Pulmonary actinomycosis is a rare, indolent infection accounting for roughly 15% of actinomycosis cases, frequently misdiagnosed as tuberculosis or malignancy owing to nonspecific clinical and radiological features. We report a 68‐year‐old male smoker with chronic alcohol use who presented with acute‐on‐chronic dyspnea, cough, and fever. Imaging revealed cavitary lesions, consolidation, ground‐glass opacities, and mediastinal lymphadenopathy suggestive of tuberculosis or malignancy. Sputum and initial microbiological workup for tuberculosis were largely negative, though a low‐level bronchoalveolar lavage GeneXpert signal prompted empirical anti‐tubercular therapy. Bronchoscopy revealed an endobronchial mass, and biopsy demonstrated pathognomonic sulfur granules composed of PAS‐positive filamentous organisms, confirming actinomycosis with no malignant cells. The patient was treated with amoxicillin‐clavulanate alongside continued anti‐tubercular therapy, with symptomatic improvement. This case highlights actinomycosis as a condition requiring a high index of suspicion when mimicking tuberculosis and lung cancer, reinforcing the necessity of histopathological confirmation for accurate diagnosis and guiding prolonged, appropriate antimicrobial management.

## Graphical abstract

A 68‐year‐old smoker with alcohol use presented with dyspnea, cough, and fever; imaging mimicked tuberculosis or malignancy. Empirical anti‐tubercular therapy was started for a low‐level GeneXpert signal, but biopsy confirmed pulmonary actinomycosis via pathognomonic sulfur granules of PAS‐positive filamentous organisms. Amoxicillin‐clavulanate with continued anti‐tubercular therapy achieved symptomatic improvement.

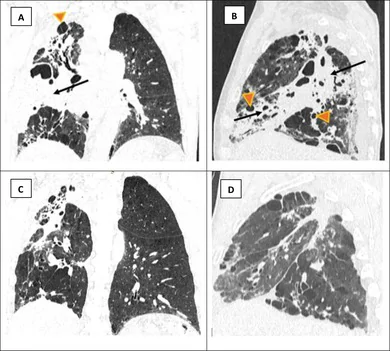

## 1 Introduction

Actinomycosis is a rare, chronic granulomatous disease caused by filamentous Gram‐positive anaerobic bacteria of the genus Actinomyces, which normally colonize the oropharynx, gastrointestinal, and urogenital tracts but become pathogenic upon invading mucosal barriers [1, 2]. Pulmonary actinomycosis (PA), accounting for ~15% of cases, results from aspiration of oropharyngeal or gastrointestinal secretions, often with dental disease or alcoholism, and may present with abscess formation, fibrosis or massive/recurrent hemoptysis requiring bronchial artery embolization [3, 4]. Due to non‐specific clinical and radiological features, PA is frequently misdiagnosed as malignancy or tuberculosis, sometimes confirmed only after surgical resection [3].

## 2 Case Report

A 68‐year‐old man, a current smoker (40 pack‐years) with chronic alcohol use (CAGE 3/4, AUDIT 9), presented with acute‐on‐chronic dyspnea (mMRC Grade I progressing to IV over 2 years, with rapid worsening over 3 days), productive cough with whitish sputum, and fever with chills. There was no hemoptysis, weight loss, or prior tuberculosis. Examination showed poor oral hygiene, tachycardia (110/min), hypoxia (SpO_2_ 75% on room air), and right infrascapular inspiratory crepitus with expiratory wheeze. He was admitted with acute infective exacerbation of COPD and Type 1 respiratory failure and was started on oxygen at 3 L/min via nasal prongs (later tapered), nebulized bronchodilators, corticosteroids, and empirical ceftriaxone and azithromycin.

Laboratory workup showed elevated inflammatory markers (CRP 173.2 mg/L, neutrophils 76.3%) and mild thrombocytopenia (platelets 81,000/μL); sputum AFB, culture, GeneXpert, and fungal stains were negative. HRCT and contrast‐enhanced CT of the chest and abdomen showed active infection superimposed on chronic lung damage, with right‐predominant cavitary lesions, mediastinal lymphadenopathy, and features of pulmonary hypertension (Figure 1A,B).

**FIGURE 1:**
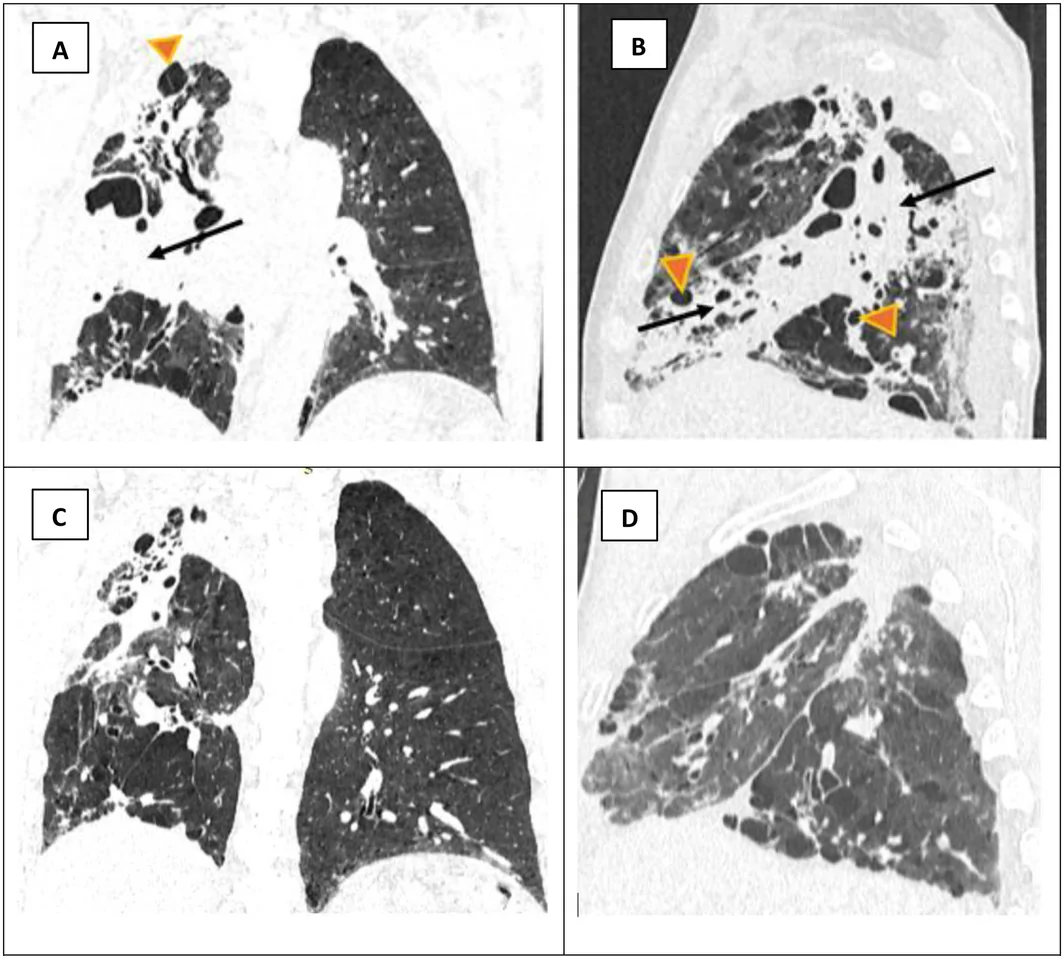
Pre‐ and post‐treatment CT chest. (A) Coronal and (B) sagittal CT lung window images at presentation show marked right lung architectural distortion with extensive cystic bronchiectasis (orange arrowheads) and associated consolidation (black arrows), predominantly involving the middle and lower lobes. (C) Coronal and (D) sagittal post‐treatment CT lung window images at 3 months demonstrate marked interval resolution of the previously extensive right‐sided consolidation.

Bronchoscopy revealed a right lower lobe endobronchial mass causing partial obstruction (Figure 2A). Bronchoalveolar lavage (BAL) GeneXpert showed very low MTB detection with indeterminate rifampicin resistance. Given the radiological and microbiological picture, empirical anti‐tubercular therapy (ATT) (HRZE) was initiated, and the patient was discharged against medical advice because of financial constraints, with bronchoscopic biopsy results pending.

**FIGURE 2:**
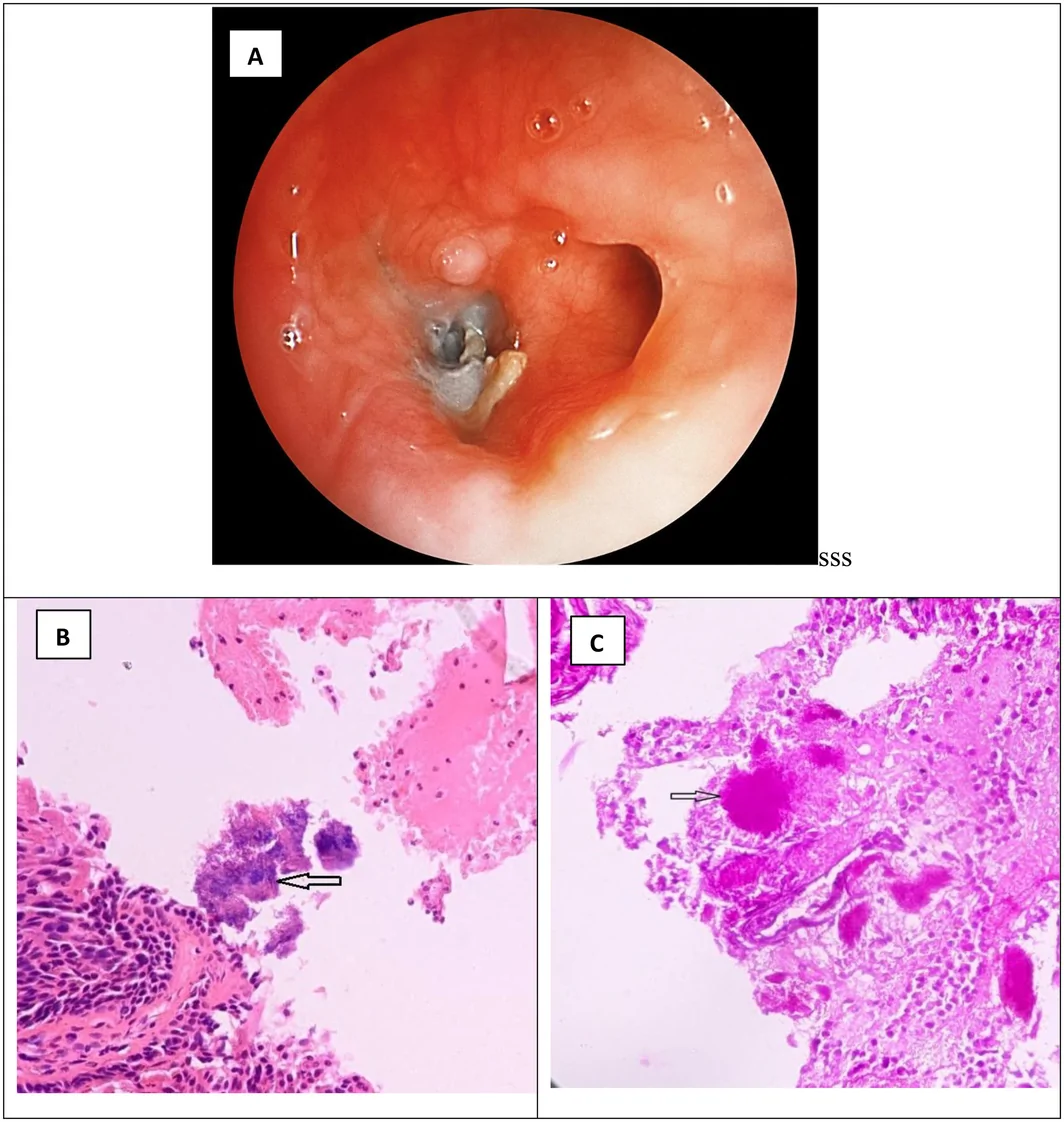
Bronchoscopic and histopathological findings. (A) Bronchoscopic view showing an inflamed bronchial tree with an endobronchial mass in the right lower bronchus causing partial obstruction of the lumen. (B) Histopathological examination of the specimen showing sulfur granules, tangled clumps of filamentous organisms (arrow). (C) Periodic Acid‐Schiff (PAS) stain highlighting PAS‐positive filamentous organisms of *Actinomyces*, exhibiting magenta positivity (arrow).

Biopsy showed suppurative, necrotic inflammation with sulfur granules of filamentous organisms and no malignant cells (Figure 2B). PAS staining highlighted the filamentous organisms, confirming pulmonary actinomycosis (Figure 2C).

The patient was started on amoxicillin‐clavulanate (1 g twice daily) alongside anti‐tubercular therapy (2HRZE/4HR). He became asymptomatic, with resolution of cough by 4 weeks. Repeat CT chest at 3 months showed marked resolution of the extensive right middle and lower lobe consolidation, indicating a favourable treatment response (Figure 1C,D), although the severe cystic bronchiectasis and architectural distortion remained unchanged, reflecting irreversible parenchymal destruction. At the time of publication, he is in the fourth month of treatment (HRZE intensive phase completed, HR continuation phase ongoing), with both amoxicillin‐clavulanate and ATT planned for 6 months in total.

## 3 Discussion

This case illustrates the diagnostic complexity of pulmonary actinomycosis, where radiological and clinical features suggested malignancy, tuberculosis, and infective COPD exacerbation, and only bronchoscopic histopathology, showing pathognomonic sulfur granules, confirmed as filamentous organisms on PAS staining, allowed a definitive diagnosis. Pulmonary involvement is the third most common form after cervicofacial and abdominopelvic disease, with peak incidence in the fourth to fifth decades and male predominance [5]. Poor oral hygiene and alcoholism are established risk factors, particularly in structurally damaged lungs, and our patient's smoking, alcohol use, and poor dentition likely created a substrate favorable for aspiration‐related infection [3].

Imaging in pulmonary actinomycosis is characteristically nonspecific, with consolidation, cavitation, ground‐glass opacity, pleural effusion, and lymphadenopathy frequently mimicking malignancy or tuberculous cavitation, as in our case [6]. Histological examination via bronchoscopic or image‐guided biopsy remains the diagnostic gold standard, as sputum or BAL culture alone is unreliable and may reflect colonization rather than true infection, explaining our patient's unrevealing microbiology [3]. Actinomyces species stain strongly with H&E, PAS, and Giemsa, but classic H&E morphology with PAS confirmation can sometimes suffice in the appropriate clinical context [3].

Anti‐tubercular therapy was initiated because of a low‐level GeneXpert signal with indeterminate rifampicin resistance, a reasonable precaution given Nepal's high TB burden. Even after histopathology confirmed actinomycosis, anti‐tubercular therapy was continued alongside amoxicillin‐clavulanate, as a low‐level positive molecular signal in a patient with cavitary disease and mediastinal lymphadenopathy could not be safely disregarded despite a negative AFB smear and culture. This dual‐therapy approach illustrates the difficulty of excluding paucibacillary or subclinical tuberculosis coexisting with actinomycosis, particularly when confirmatory culture data remain pending.

Actinomyces species are highly susceptible to beta‐lactams, with penicillin or amoxicillin as first‐line therapy for 1–2 months; surgery is reserved for complicated cases such as critical‐space infection, massive hemoptysis or medical treatment failure [3]. In our patient, amoxicillin‐clavulanate was added empirically alongside continued anti‐tubercular therapy, reflecting diagnostic uncertainty rather than a stepwise substitution of one diagnosis for another.

This case's principal strength is its layered diagnostic reasoning when radiology alone is nonspecific, with histological, radiological and clinical data triangulated toward a defined management plan. Its limitations include the lack of species‐level microbiological confirmation.

Pulmonary actinomycosis remains a diagnostic chameleon requiring a high index of suspicion whenever a presumed malignant or tuberculous lesion behaves atypically. In endemic settings, concurrent management of actinomycosis and possible tuberculosis may be warranted until confirmatory testing excludes coinfection.

## Author Contributions

**Safal Bogati:** conceptualization, investigation, methodology, writing – review and editing, writing – original draft, supervision, validation. **Bishal Pradhan:** investigation, writing – original draft, writing – review and editing, visualization, methodology, conceptualization, validation. **Suraksha Thapa:** validation, writing – original draft. **Milesh Jung Sijapati:** conceptualization, supervision, validation. **Prabhas Joshi:** validation, writing – original draft, investigation, formal analysis. **Richa Ghimire:** validation, investigation, formal analysis. **Samikchhya Regmi:** validation, investigation.

## Funding

The authors have nothing to report.

## Consent

The authors declare that written informed consent was obtained for the publication of this manuscript and accompanying images using the consent form provided by the Journal.

## Conflicts of Interest

The authors declare no conflicts of interest.

## Data Availability

The data that support the findings of this study are available from the corresponding author upon reasonable request.
